# Design and Fabrication of Low-Cost 1536-Chamber Microfluidic Microarrays for Mood-Disorders-Related Serological Studies

**DOI:** 10.3390/s131114570

**Published:** 2013-10-28

**Authors:** Xinyan Zhao, Tao Dong

**Affiliations:** Department of Micro and Nano Systems Technology (IMST), Faculty of Technology and Maritime Sciences (TekMar), Vestfold University College (HiVE), Tønsberg, N3103, Norway; E-Mail: Xinyan.zhao@hive.no

**Keywords:** microfluidic microarray, lab on a chip, chemiluminescent immunoassay, carbon dioxide laser ablation, carbon dioxide laser ablation, mood disorder

## Abstract

Mood disorders are common mental diseases, but physiological diagnostic methods are still lacking. Since much evidence has implied a relationship between mood disorders and the protein composition of blood sera, it is conceivable to develop a serological criterion for assisting diagnosis of mood disorders, based on a correlative database with enough capacity and high quality. In this pilot study, a low-cost microfluidic microarray device for quantifying at most 384 serological biomarkers at the same time was designed for the data acquisition of the serological study. The 1,536-chamber microfluidic device was modeled on a 1,536-well microtiter plate in order to employ a common microplate reader as the detection module for measuring the chemiluminescent immunoassay tests on the chips. The microfluidic microarrays were rapidly fabricated on polymethylmethacrylate slides using carbon dioxide laser ablation, followed by effective surface treatment processing. Sixteen types of different capture antibodies were immobilized on the chips to test the corresponding hormones and cytokines. The preliminary tests indicated that the signal-to-noise ratio and the limit of detection of microfluidic microarrays have reached the level of standard ELISA tests, whereas the operation time of microfluidic microarrays was sharply reduced.

## 1. Introduction

Previous studies have shown that mood disorders are related to changes in the profile of signaling molecules in blood [1,2]. Steiner *et al.* proposed a biological susceptibility hypothesis to account for gender differences in the prevalence of mood disorders, based on the idea that there is a disturbance in the interaction between the hypothalamic-pituitary-gonadal axis and other neuromodulators in women [3]. According to this hypothesis, the neuroendocrine rhythmicity related to female reproduction is not only vulnerable to change, but also sensitive to psychosocial, environmental and physiological factors [3]. In the light of the explosion in psychiatric neuroscience research in the past decade, some consensus regarding significant problems in neuropsychopharmacology has been reached [4,5]. For unipolar and bipolar disorder, however, there have been very few significant innovations and no genuine breakthrough drugs in the past two decades [5,6]. The primary focus of past and current research into mood disorders has been the biology and neural circuitry most relevant to the monoaminergic systems, *i.e.*, serotonin, norepinephrine (NE) and dopamine [6–8].

As an integral part of the stress response system of organisms, NE serves as an essential neurotransmitter that regulates arousal and adapts to environmental and internal stressors [7]. The central NE system is closely related to common mental diseases, such as anxiety, depression and panic disorders [7,9,10]. According to the past studies, the NE system participates directly in the development of anxiety and depression [7]. For example, stress is first applied in the nervous system and then affects the endocrine system. The acute stress response is illustrated by stressors activating the hypothalamic-pituitary-adrenal axis and the LC-NE pathways, which results in the release of stress hormones from the paraventricular nucleus and dorsomedial hypothalamus [7]. Although it is known that the central NE system interacts with the hypothalamic-pituitary-adrenal system to influence mood disorders, the underlying neurobiological mechanisms involved are still not well understood [7,11,12]. Consequently, it is generally believed that there is a mapping relationship between the profile of signaling molecules in blood and mood disorders.

Blood testing is the most common examination performed in hospitals. The number of detection items in a single blood test typically ranges from several to tens. There are around four hundred types signaling molecules in the blood stream known at present, whereas the common referenced ones in a hospital are less than fifty. Although they are only minority components of blood, their functions are more crucial than those of others. Low-throughput antibody microarrays based on immunoassay reliably acquire signal-molecule-profiling (SMP) data from sera samples (e.g., Human Cytokine Microarray; Allied Biotech, Inc©, Ijamsville, MD, USA). The detection limit of on-chip immunoassays, combined with proper equipment, has been able to cover the needs of SMP data acquisition. However, present protein microarrays are too expensive to be practical tools to quantify hormones and cytokines. The protein microarrays have been highly developed for more than 10 years [13], but they still present some disadvantages. First, the present microarrays are often built on a flat glass slide or matrix-based material. The reaction interface on the slide might be easily smeared when manipulated carelessly. Second, due to the slow speed of molecular diffusion, complete bio-hybridization assays usually require several hours, or even one day. Lastly, the flat structures of microarrays might result in overlapped signals between amplification reactions of adjacent spots. Wang *et al.* reported a CD-like microfluidic microarray device for the rapid discrimination of fungal pathogenic DNA, but it is hardly compatible with common devices used for previous microarray chips [14]. The demand of the serological study calls for low-cost ultrasensitive tools. In this study, a microfluidic microarray with three-dimensional microfluidic structures was developed, which resolves the shortcomings of previous microarrays and could employ the research and development (R&D) systems developed for common microarrays, such as Flexible Annotation and Correlation Tool [15] and AD1500 R&D System (Biodot©, Irvine, CA, USA). The reactive interfaces are located inside chambers, allowing the three-dimensional structures to protect the crucial surfaces, but the chamber arrays are distributed in a flat chip, similar to ordinary microarray chips. The narrow space in reaction chambers can limit the diffusion distance such that the efficiency of bio-hybridization could be enhanced and the total operation time may be shortened in theory. Moreover, isolated chambers offer a wide selection of amplification reactions, such as immuno-PCR, immuno-NASBA and chemi-luminescent immunoassay (CLIA) *etc.*, which are all highly ultrasensitive detection methods characterized by femtomolar sensitivity and high specificity [16–18]. Here, the mature method CLIA was selected for the microfluidic microarray chips.

The serological tests on a microfluidic microarray chip can be regarded as an extended blood examination that aims to measure hundreds of the most important biomarkers in the blood, *i.e.*, cytokines and hormones. Given that the results of microarray tests can cover the common information of regular blood tests, partial results of microarray tests can be accepted by doctors theoretically. Furthermore, these records are good resources for data mining in the field of translational bioinformatics. The hidden information in a serological profile will be used to evaluate the physical conditions of individual patients in the future. The database generated by the low-cost microarrays is both an excellent resource for scientists and a compatible tool for doctors in hospitals. In fact, there is no mathematical challenge to develop some serological criteria for assisting diagnosis for mood disorders, as long as a qualified serological database of patients with mood disorders could be established. The low-cost tool for data acquisition is the key to the serological study, which is the focus of this work.

## 2. Experimental Section

### 2.1. Chip Design

**Figure 1.**
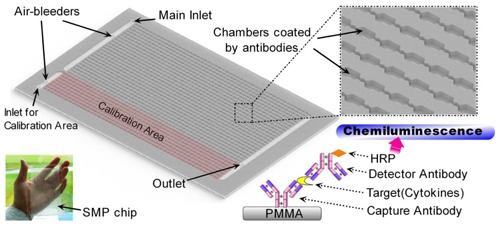
Chip layout of 1536-chamber microfluidic microarray (**Top**) Inlet and outlet ports, chambers, and the calibration area of 1536-chamber chip were diagramed in the middle. (**Bottom right**) The detection principle of sandwich-type CLIA was shown at the bottom right corner. (**Bottom left**) A photo of the chip prototype was shown at the bottom left corner.

Low-cost materials and fabrication methods were employed to reduce the fabrication cost. Polymethylmethacrylate (PMMA), a common bio-compatible material, was selected to make the microfluidic devices. Besides, PMMA is suitable for both low-cost fabrication methods, *i.e.*, carbon dioxide laser ablation in the R&D stage and injection molding in the next stage of mass production. Since the number of detection targets, hormones and cytokines are no more than 400, the structure of high-density microarray is not necessary in this case. Therefore, the 1,536-chamber microfluidic microarray chip called “SMP chip” was designed (Figure 1). It can standardize the simultaneous detection of at most 384 types of signaling molecules in one blood sample, while every detection item takes three parallel measures. Similar to the previous immuno-NASBA chips, the SMP chip has the same dimensions as the 1,536-well microtiter plate, thus making the SMP chip readable in a microplate reader [16,19].

The SMP chip can be considered a low-throughput protein microarray with a 3D structure. The reaction interface inside the chambers is protected by a stereo structure, and the narrow space of the chambers can enhance the efficiency of the immunoassay. In consideration of the chosen material and method for fabrication, the cost of SMP chips did not increase significantly compared with that of a planar microarray. Moreover, the compatibility of the SMP chip brings multiple choices of detection modules. The detection module here was a microplate reader in a common biological laboratory, whereas the specific chip reader in the future could be easily updated from that classic machine. The current instruments for handling ordinary microtiter plates could also turn into instruments for the surface treatment of SMP chips. Thus, the difficulties in R&D of the SMP chip and its detecting system could be reduced.

In the SMP chip, the area of 384 chambers was preserved for calibration tests, while 1,152 chambers were left for monitoring 384 kinds of targeted molecules in one serum sample. The SMP chip, 127.8 mm in length, 85.5 mm in width and 2 mm in height, was fabricated in a laminated PMMA slide on the carbon dioxide laser engraving machine (Feilijia^®^ H4030, Liaocheng, China) [20]. Every chamber is around 1.1 mm in length, 1 mm in width and 0.4 mm in depth, which volume is about 4.4 × 10^−1^ m^3^. The sensing capability of the chip depends on the quality of surface treatment and the performance of CLIA system.

### 2.2. Surface Treatment on PMMA

**Figure 2.**
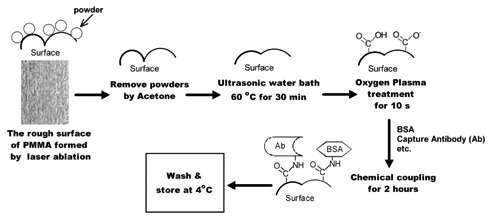
The flow diagram of surface modification processing on the microfluidic microarray.

After the SMP chip was made by carbon dioxide laser ablation, its PMMA surface in chambers were rather rough and often covered by powders, as shown in Figure 2. The surface roughness in every chamber should be decreased before capture antibodies are immobilized. The surfaces of PMMA prototypes were rinsed with acetone in order to remove adhered powders. After the evaporation of the acetone, the PMMA prototypes were incubated in an ultrasonic water bath (about 60 °C) for half an hour. The post-exposure heat treatment might partially melt the PMMA surface with the help of ultrasonic energy, and thus form a smoother surface instead. After that, the PMMA surface was modified by oxygen plasma treatment. Commercially available oxygen was released into the discharge chamber. After the oxygen plasma was created, the primary prototypes were exposed in the plasma for 10 s. Then, capture antibodies were chemically coupled onto the PMMA surface by the use of EDC (1-ethyl-3-(3-dimethylaminopropyl) carbodiimide) reagent (ShenYuan ChemPharm^®^, Xiao Gan, China) [21]. The solution for chemical coupling was composed of 70 μL of 2-(N-morpholino) ethanesulfonic acid buffer (MES, Li Tian Sci. &Tech^®^, Wuhan, China), 10 μL of EDC reagent, 2 μL capture antibody stock solution (0.4 mg/mL) and 2 μL bovine serum albumin (BSA) solution (2 mg/mL, Li Tian Sci.& Tech^®^). Each mixed solution was manually added into the corresponding chambers by a 2 μL pipette with the help of a printed grid under the PMMA chip. The corresponding location of chambers on the printed grid was marked by different colors for operators to target the location of different antibodies. The loaded solution in PMMA chambers would incubate for about 2 h.

After that, the chambers were washed by phosphate buffered saline (PBS) solution, and coated by SuperBlock^®^ (PBS) blocking buffer (Thermo Scientific^®^, Rockford, IL, USA) for 12 h. Transparent sealing tape (Biovendis^®^, Mannheim, Germany) was used as the cover layer on the treated PMMA layer. Complete microfluidic microarrays were stored at 4 °C before use. The relevant holes for the inlet, outlet and air bleeder have been ablated through the PMMA layer by laser before the surface treatment process. The capability of the SMP chip was not completely exploited at the pilot stage. To demonstrate the function of microfluidic microarray chip, 16 types of different monoclonal antibodies were immobilized on the chip to measure 16 types of signaling molecule targets, including thyroid-stimulating hormone (TSH), tumor necrosis factor-alpha (TNF-α), cortisol, follicle-stimulating hormone (FSH), luteinizing hormone (LH), human chorionic gonadotropin (HCG), erythropoietin (EPO), gonadotropin-releasing hormone (GnRH), interferon-gamma (IFNγ), insulin-like growth factor 1 (IGF-1), epidermal growth factor (EGF), vascular endothelial growth factor (VEGF), interleukin (IL)-1 beta, IL-2, IL-6 and IL-8. These capture antibodies and respective standard samples of antigens came from Li Tian Sci. &Tech Co. Ltd.

### 2.3. Protocols of CLIA Tests on the Microfluidic Microarray

The sandwich-type CLIA was employed on the microfluidic microarray shown in Figure 1. The immunoassay took place on the PMMA surface inside the reaction chamber. All reagents were prepared using sterile deionized water. The surface of the reaction chambers were coated with capture antibodies, while the blocking buffer covered the remaining residual absorptive sites. The parallel reactions in 1,536 chambers of one chip are operated through an external valve and pump system, which the typical protocol of the CLIA test is listed in Table 1. The total operation time was about 1/2 h. Since the microfluidic channels on the SMP chip are wide enough, 1 × 10^−7^ m^3^/s did not exceed the capability of those channels. The high flow rate in Table 1 could result in a short operation time. Besides, the minimum volume of samples for a typical SMP test is about 1 mL.

After initial washing, the chambers were incubated with sample solutions. As described above, 384 chambers, 1/4 area of the microfluidic microarray were designed for calibration tests. One kind of standard solution with known artificial antigens was loaded into these calibration chambers, meanwhile, the sample solution was loaded into the other 1,152 chambers through the main inlet. The next steps for all chambers in one chip were synchronized. After every kind of target biomarker was captured in the respective chamber, unbound molecules would be washed away by PBST and PBS solutions. Then, the chambers were filled by the solution of detector antiserum-HRP conjugates, which antiserum was obtained from immune mice via traditional methods. After incubation, the sandwich-type immune complexes would be formed on the PMMA surface. Finally, the working solution of SuperSignal^®^ ELISA femto maximum sensitivity substrate (Thermo Scientific^®^, Rockford, IL, USA) was introduced into the chambers within 10 seconds. When the SMP chip was disassembled from the external valve and pump system, it would be quickly moved into a ready microplate reader that had been preheated to 300 K. The chemiluminescent data of all chambers would be read in 5 min.

TNF-α was employed as a model to test the calibration tolerance of the SMP chip. Five standard solutions of TNF-α were serially diluted in PBS with 5% BSA, and then quantified using the SMP chips. The relative luminescence units (R.L.U.) data of chambers were collected by the microplate reader and analyzed using Microsoft Excel^®^ and OriginPro^®^. 42 clinical serum samples of Chinese women were provided by our medical partner. These clinical samples were measured by the SMP chips, and they were also tested by the medical partner through traditional ELISA methods. The testing results were shared and compared in OriginPro^®^. Moreover, several roughcast SMP chips were also made by laser ablation and then immobilized with capture antibodies through physical adsorption. The batch of roughcast SMP chips and the complete SMP chips were both examined using a standard solution of TNF-α and one clinical serum sample, in order to evaluate the effectiveness of surface treatment steps.

## 3. Results and Discussion

### 3.1. Calibration Tests for Artificial Samples

**Figure 3.**
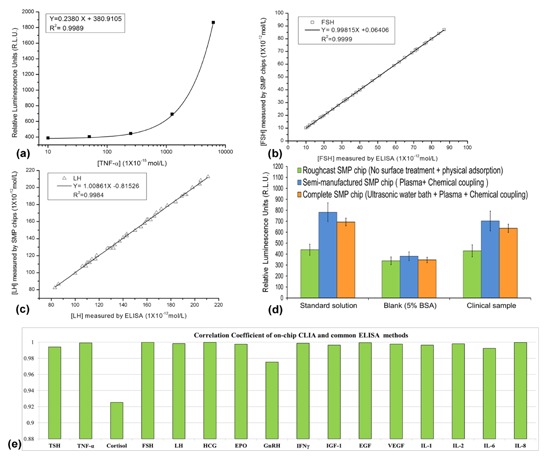
Quantitative detection assays on the microfluidic microarrays. (**a**) The calibration curve of TNF-α; (**b**) Comparison plot for FSH concentration of 42 clinical samples by using both the SMP chips and traditional ELISA method; (**c**) Comparison plot for the concentrations of LH measured in 42 clinical samples using both the SMP chips and traditional ELISA method; (**d**) Comparison of the R.L.U. value in the CLIA tests for TNF-α. Three kinds of SMP chips, the complete SMP chips, the semi-manufactured SMP chips and the roughcast chips without surface treatment, were employed. The standard solution here was the TNF-α solution of 1.25 × 10^−12^ mol·L^−1^; the concentration of TNF-α in the clinical sample here was about 1.07 × 10^−12^ mol·L^−1^; (**e**) All correlation coefficient factors of 16 comparison plots for corresponding biomarkers in the 42 clinical samples by using both the SMP chips and traditional ELISA method.

Five kinds of TNF-α standard solutions were tested on the SMP chips, which concentrations were 1 × 10^−14^, 5 × 10^−14^, 2.5 × 10^−13^, 1.25 × 10^−12^ and 6.25 × 10^−12^ mol·L^−1^. The blank control was only 5% BSA in PBS solution. Through the standard operating procedures in Table 1, the R.L.U. data of SMP chips were measured and diagrammed as Figure 3a, in which a typical linear calibration curve was demonstrated. When the concentrations of TNF-α are over 5 × 10^−14^ mol·L^−1^, it can be precisely quantified. The standard solution of 1 × 10^−14^ mol·L^−1^ only show a weak positive signal, close to the blank control, which indicated that the limit of detection (LOD) of the SMP chip is around 1 × 10^−14^ mol·L^−1^. This LOD has reached the typical detectability of a common ELISA test, which had been verified by manual CLIA tests. The standard curves for the other 15 cytokines and hormones were made by the same method as the experiments of TNF-α.

### 3.2. Validation of the SMP Chips Using Clinical Samples

Normalized measurement of on-chip CLIA tests could provide comparative data as the traditional ELISA methods. The typical comparison plots of two hormones from both SMP chip testing and ELISA are shown in Figure 3b,c. FSH and LH in 42 clinical samples were measured. The 1:10 dilutions of clinical samples with PBS were always made before loading.

Although it is not necessary to dilute the samples, the step of dilution could reduce the blood volume required for each SMP test and standardize the pH of sample solutions for antibody-based assays. The robust capability of CLIA detection often covers the concentration range of 5 or 6 orders of magnitude [22,23], so the dilution ratio of 1:10 will not hinder the measurement for most physiological or pathological concentrations of cytokines and hormones in human blood. However, the dilution factor should be fixed in advance because the optimization of surface treatment and standard protocols of the SMP chips is related to that factor.

From the results in Figure 3, we found the differences of both results were extremely small, which implied that the correctness and precision of the SMP chip was verified as good as traditional ELISA tests. The comparison plots of other 14 hormones were similar to Figure 3b,c, whereas their correlation coefficient factors of plots were varied. All 16 kinds of on-chip measuring systems for 16 hormones and cytokines showed similar capability of quantifying biomarkers as common ELISA systems (Figure 3e). Most correlation coefficient factors between measuring results from two methods were over 0.99, whereas the correlation coefficient factors for comparison plots of Cortisol and GnRH tests were obviously lower than others. Based on our experience, this result might be caused by the lower quality of capture antibodies or the instability of the target antigens. The quality of capture antibodies is always the critical factor to the precision of an immune-detection system.

### 3.3. Evaluation of Surface Treatment Processing

The surface treatment processes for the SMP chip were more complex than previous 6-channel microtiter chips, which were also made of PMMA [16]. The surfaces of PMMA chambers after the ultrasonic heat treatment were examined by using a microscope and a surface profile gauge. It was verified that the treatment was able to make the surfaces of the PMMA chamber smoother. Even the transparency of the micro-structures on PMMA chips can be improved. The purpose of this treatment is to standardize the area of surfaces in chambers, while the uniform chambers could decrease the background noise of the measurement. On the other hand, the consecutive processes, *i.e.*, oxygen plasma and chemical coupling aimed to increase the density of capture antibodies on the chamber surfaces so as to enhance the signal of CLIA tests in chambers. Fortunately, the chemical coupling process will not increase nonspecific adsorption greatly. Therefore, the signal-to-noise ratio (SNR) could be increased. Both processes of ultrasonic heat treatment and chemical coupling contribute towards the improvement of SNR. However, these processes cannot replace each other due to their distinct principles. Briefly, the ultrasonic heat treatment is to reduce the difference in parallel chambers, whereas the chemical coupling aims to directly improve SNR of CLIA tests in every chamber.

To evaluate the effectiveness of surface treatment steps, the roughcast SMP chips without surface treatment were specially prepared for the comparison tests. Besides, the semi-manufactured SMP chips without ultrasonic heat treatment but with chemical coupling were also made for the same purpose. Three samples were tested on all three kinds of chips, which results were shown in Figure 3d. For the blank control, no significant difference was found between the results from three kinds of chips. However, the R.L.U. signals in the complete SMP chips were much higher than those in the roughcast chips, when the artificial solution of TNF-α and the clinical sample were employed. The results of semi-manufactured SMP chips were similar to those of the complete SMP chips, but the mean and variance of the former results were obviously lager than the latter ones. In addition, a few SMP chips only treated by ultrasonic heat treatment were also tested, which results were as similar as the roughcast SMP chips. Therefore, the chemical coupling seems to be more important than ultrasonic heat treatment, but the latter step could improve the precision of measurement. The signal-to-noise ratio (SNR) of the complete SMP chips was higher than that of the chips without surface treatment. It seems that more capture antibodies were immobilized on the PMMA surface through the method of chemical adsorption than only using physical adsorption. The common ELISA tests often employ physical adsorption methods to immobilize proteins. Although the steps of oxygen plasma and chemical coupling were time-consuming, the surface modification processing could improve the SNR of the PMMA chips to the similar level as traditional ELISA methods. Otherwise, the shortcoming of laser ablation method often results in low sensitivity of the prototype chips.

### 3.4. Discussion on the Low-Cost Fabrication Method

As a low-cost microfluidic device, the tolerances of low-cost fabrication methods have no comparability with precision machining technologies [24], such as bulk silicon micromachining technique on the microfluidic systems developed in Høgskolen i Vestfold^®^ [25–27]. One compromise strategy is to enlarge the chip size, considering cheap materials are often employed. However, the methods of surface treatment have to be designed carefully, because the accuracy of on-chip measurement is nearly determined by the surface of chambers. Without precise ability of detection, the low-cost microfluidic chips will be worthless. The method of carbon dioxide laser ablation is popular in the fabrication of low-cost microfluidic devices; however, the coarse surface formed by laser ablation usually limits its application [28]. In this study, series of surface treatment processes were selected and performed after laser writing, which could partially remedy some defects on the PMMA surface. Because of the surface treatment process, the 1536-chamber microfluidic device has enough sensitivity and good precision in the following tests. The compatibility of SMP chips to common microplate readers would also help users to easily accept the SMP chips as blood-test tools. Although 16 capture antibodies could be handled manually in this study, 384 kinds of different capture antibodies in the future were impossible to be handled manually in the surface treatment of SMP chips. Some existing robot systems for common microarray chips will be employed to distribute different antibodies into corresponding chambers. The compatibility of the SMP chip is not only limited to its detection module, but also to the existing instruments for its surface treatment.

The SMP chip has 1,536 chambers, but the chambers are separated into two sections for two solutions. One is designed for testing a serum sample, the other section is left for the test of a standard solution, which is called as “calibration area” in Figure 1. Apparently, a single SMP chip could not obtain enough data for a calibration curve. This might be a shortcoming of the SMP chip, but the problem should disappear when the SMP chips become widely used. If more than 16 serum samples are tested, 16 SMP chips would allow 16 parallel calibration tests using different standard solutions. Thus, the batch of standard SMP chips can share one collective calibration curve, which is also regarded as a low-cost way to obtain a standard curve. In fact, any single microarray chip is not insufficient to measure a sample as well. Usually, a series of standard solutions for calibration curves have to be previously measured by additional microarray chips. Due to different oriented users, the SMP chips were designed for the large-scale routine testing in hospitals, instead of a small-range scientific study. The data for standard curves could be accumulated in pace with the SMP-chip tests for patients, which number would easily exceed the minimum amount of SMP chips to obtain a batch of calibration curves. Furthermore, the more patients use the SMP chips, the more data will be collected for the correction of standard curves, which lead to better reliability of the measurement. If the SMP chips have to be employed in a small-scale test, some additional SMP chips with all 1,536 chambers could be used to make standard curves, resembling other microarray chips.

## 4. Conclusions/Outlook

The SMP chip was specially designed for the data collection of a mood-disorders-related serological database. The chip was required to be cheap but precise enough, which has been preliminarily validated by the study. Being a typical device of a database-oriented lab-on-a-chip, the microfluidic microarray was fabricated by low-cost processing methods, but its ability of detection was as good as ordinary ELISA methods. In the future, such database-oriented lab-on-chip devices might grow into a new branch in the microsystems research field. Compared with the study for MicroTAS systems [29], which try to make the microdevices more and more complex [30], the database-oriented lab-on-chips will be simpler and simpler with the essential precondition of qualified detection capability, so as to reduce the cost of data acquisition in the medical investigation. This study built a model for other database-oriented lab-on-a-chip devices.

The serological-markers-based diagnosis on mood disorders is a magnificent challenge that resembles the Human genome project. The diagnosis method is feasible; however a qualified database has to be established initially. In the past decades, many doctors and scientists tried to find out the diagnosis relationship by using limited clinical trials, but unfortunately, no breakthrough was made. It is widely believed that that relationship might be rather complex, which only biometricians or mathematicians were able to describe it with an accurate format. Before the breakthrough, the scientists have to develop a low-cost tool for widely collecting human serological information, and then accumulate a large-scale database for biometricians and mathematicians. The whole project will be a systematic engineering with interdisciplinary cooperation.

## Figures and Tables

**Table 1.** The typical protocol of on-chip CLIA tests using the microfluidic microarray device.

**Steps**	**Description (Room Temperature** = **300 K)**	**Parameters**
1. Rinse chambers	Successively rinse the channels and chambers of the SMP chip by PBST (Phosphate Buffered Saline with 0.05% Tween^®^-20) and PBS by syringe pumps	Flow rate: 1 × 10^−7^ m^3^/s Duration: 30 s per solution
2. Loading sample	Load a standard solution into the inlet of calibration area; load the serum sample at the same time into the main inlet	Flow rate: 1 × 10^−7^ m^3^/s Duration: 10 s
3. Incubation	Incubate the SMP chip at room temperature for 10 min	Flow rate: 0 m^3^/s Duration: 600 s
4. Rinsing chambers	Repeat Step 1	Flow rate: 1 × 10^−7^ m^3^/s Duration: 30 s per solution
5. Loading detector antibodies	Load the solution containing detector antiserum-HRP conjugates	Flow rate: 1 × 10^−7^ m^3^/s Duration: 10 s
6. Incubation	Incubate the SMP chip at room temperature for 5 min	Flow rate: 0 m^3^/s Duration: 300 s
7. Rinsing chambers	Repeat Step 1	Flow rate: 1 × 10^−7^ m^3^/s Duration: 30 s per solution
8. Loading substrate	Load the working solution of SuperSignal^®^ ELISA Femto Maximum Sensitivity Substrate(Thermo Scientific^®^, Rockford, IL, USA)	Flow rate: 1 × 10^−7^ m^3^/s Duration: 10 s
9. Measuring R.L.U. in a microplate reader	Move the chip into a microplate reader quickly, and read relative luminescence units (R.L.U.) of every chamber in 5 min	Duration: 300 s
		Total operating time: 1,450 s (approx.)
